# Prenatal Glucocorticoid-Exposed Infants Do Not Show an Age-Typical Fear Bias at 8 Months of Age – Preliminary Findings From the FinnBrain Birth Cohort Study

**DOI:** 10.3389/fpsyg.2021.655654

**Published:** 2021-07-28

**Authors:** Eeva-Leena Kataja, Ana João Rodrigues, Noora M. Scheinin, Saara Nolvi, Riikka Korja, Tuomo Häikiö, Eeva Ekholm, Nuno Sousa, Linnea Karlsson, Hasse Karlsson

**Affiliations:** ^1^The FinnBrain Birth Cohort Study, Turku Brain and Mind Center, Department of Clinical Medicine, University of Turku, Turku, Finland; ^2^Department of Psychology and Speech-Language Pathology, University of Turku, Turku, Finland; ^3^Life and Health Sciences Research Institute (ICVS), School of Medicine, University of Minho, Braga, Portugal; ^4^Department of Psychiatry, Turku University Hospital, University of Turku, Turku, Finland; ^5^Department of Psychology and Speech-Language Pathology, Turku Institute for Advanced Studies, University of Turku, Turku, Finland; ^6^Department of Medical Psychology, Corporate Member of Freie Universität Berlin, Berlin Institute of Health (BIH), Humboldt-Universität zu Berlin, Charité – Universitätsmedizin Berlin, Berlin, Germany; ^7^Department of Obstetrics and Gynecology, Turku University Hospital, University of Turku, Turku, Finland; ^8^Centre for Population Health Research, Turku University Hospital, University of Turku, Turku, Finland

**Keywords:** synthetic glucocorticoids, eye tracking, emotion processing, attention, fear bias, prenatal

## Abstract

Synthetic glucocorticoids (sGC) are frequently administered to pregnant women at risk for preterm delivery to promote fetal lung maturation. Despite their undeniable beneficial effects in lung maturation, the impact of these hormones on developing brain is less clear. Recent human studies suggest that emotional and behavioral disorders are more common among sGC-exposed vs. non-exposed children, but the literature is sparse and controversial. We investigated if prenatal sGC exposure altered fear bias, a well-established infant attention phenotype, at 8-months. We used eye tracking and an overlap paradigm with control, neutral, happy, and fearful faces, and salient distractors, to evaluate infants’ attention disengagement from faces, and specifically from fearful vs. neutral and happy faces (i.e., a fear bias) in a sample (*N* = 363) of general population from the FinnBrain Birth Cohort Study. sGC exposed infants (*N* = 12) did not differ from non-exposed infants (*N* = 351) in their overall probability of disengagement in any single stimulus condition. However, in comparison with non-exposed infants, they did not show the age-typical fear bias and this association remained after controlling for confounding factors such as prematurity, gestational age at birth, birth weight, sex, and maternal postnatal depressive symptoms. Prenatal sGC exposure may alter emotional processing in infants. The atypical emotion processing in turn may be a predictor of emotional problems later in development. Future longitudinal studies are needed in order to evaluate the long-term consequences of sGC exposure for the developing brain.

## Introduction

Maternal stress hormones, glucocorticoids (GC), are critical to normal fetal development, increasing adaptively in concentration over the human pregnancy to ensure fetal maturation and to prepare for birth (Mastorakos and Ilias, 2003). GC are potent hormones with pleiotropic physiological effects, so the levels to which the fetus is exposed are tightly controlled by placental inactivation of active GC (cortisol in humans, corticosterone in rodents) to metabolites by the enzyme 11β-hydroxysteroid dehydrogenase type 2 (11β-HSD2) (Seckl and Holmes, 2007). This rigorous control is important for normative fetal development, as abnormal levels of these hormones can increase the risk for adverse neurodevelopment and health outcomes later in life.

According to WHO, around 15 million babies are born preterm every year (10% of all live births) (Blencowe et al., 2012). Preterm birth is a major cause of neonatal mortality and morbidity worldwide (Teune et al., 2011; Liu et al., 2016). Synthetic glucocorticoids (sGC) are widely used during pregnancies with risk of premature delivery to promote fetal lung maturation and to prevent respiratory distress syndrome in preterm infants. In high-income countries, most of the women at risk of preterm birth receive synthetic corticosteroids, in contrast with far less in middle and low-income countries. In a cross-sectional survey database of birth outcomes in 29 countries, the rate of sGC use varied between 16 and 91% (Vogel et al., 2014). Current clinical guidelines indicate that mothers at risk of premature delivery before 34 weeks of gestation are candidates for prenatal sGC therapy. Nowadays, the recommended treatment courses include one single course of sGC, consisting of two doses of 12 mg betamethasone administered intramuscularly 24 h apart or four doses of 6 mg dexamethasone administered intramuscularly every 12 h. sGC such as beta- and dexamethasone are commonly preferred because they are not metabolized by placental 11β-HSD2, and thus reach the developing fetus in supraphysiological levels (Kajantie et al., 2004), accelerating maturation of the preferred tissues. These sGC are also more potent than endogenous cortisol and bind to glucocorticoid receptors with more affinity than endogenous GC that preferentially bind to mineralocorticoid receptors (Axelrod, 1976). This leads to a differential molecular response and more potent effects of sGC.

However, although a consensus is solid in that the administration of sGCs has decreased neonatal mortality and morbidity, and their benefits outweigh the potential longer-term harms sGC may carry on individuals born preterm (National Institutes of Health Consensus Development Panel, 2001), there is still the need to perform additional studies to understand the long-term impact of prenatal sGC administration. Animal studies have shown that prenatal sGC exposure alters the HPA axis and leads to structural and functional changes in several different brain regions, including the prefrontal cortex and limbic regions important for emotion processing and regulation such as the hippocampus, nucleus accumbens, and amygdala, among others (McArthur et al., 2005; Oliveira et al., 2006, 2011, 2012; Rodrigues et al., 2012; Reynolds, 2013; Cartier et al., 2016). Considering these alterations, it is not surprising that a large number of studies suggest that prenatal sGC-exposed animals present with emotional and behavioral deficits, including anhedonia and depressive-like behavior (Oliveira et al., 2006; Soares-Cunha et al., 2014; Coimbra et al., 2017), anxiety (Oliveira et al., 2006; Duarte et al., 2019) as well as increased addictive behaviors (Rodrigues et al., 2012).

In humans, there is clear evidence that prenatal sGC exposure leads to decreased birth weight, even in term born babies (Rodriguez et al., 2019). In addition, accumulating evidence suggests that prenatal sGC exposure may be associated with emotional and behavioral problems later in life. For example, in a large cohort of >4,500 mother-children dyads, the prevalence of any mental, emotional and behavioral disorder was higher in the sGC-exposed group when compared to non-exposed children (Wolford et al., 2019). Mothers of sGC-exposed children also reported that their children had more psychiatric problems and higher risk of not meeting age-appropriate developmental and personal-social skills, independently of prematurity. A more recent study investigated similar effects in a retrospective cohort of >670,000 children using nationwide registries. Treatment exposure, compared with non-exposure, was significantly associated with higher risk of any mental and behavioral disorder, and more strongly among children born at term (Räikkönen et al., 2020). One study also found that children (6–10 years) with fetal sGC exposure had prominent cortical thinning in the right anterior cingulate cortex (rACC), though they did not manifest significant affective problems at that age. Yet, because a thinner rACC is associated with risk for affective problems, authors postulated that this phenotype could arise later in life (Davis et al., 2013).

Conversely, some studies have found no association between prenatal sGC and later child outcomes. One randomized controlled trial found no differences in hyperactivity, emotional symptoms, prosocial behavior, conduct, or peer problems between the treated and non-treated children (Stutchfield et al., 2013). Two other studies found no differences in the levels of affective problems or intelligence (Davis et al., 2013; Alexander et al., 2016). These controversies may result from diverse study designs, differences in outcome definitions, and measurements as well as differences in the type, number of courses, doses, and timing of sGC administration. In addition, it is difficult to disentangle direct effects of sGC from confounding variables such as prematurity and maternal and child health conditions. However, based on previous literature on the effects of sGC on child’s emotion and behavior regulation and the knowledge that the developing emotion processing systems, and specifically fear systems, are highly sensitive to the influences of cortisol, possibly already in prenatal life (Cartier et al., 2016; Callaghan et al., 2019), these developing emotion processing systems are important targets of study.

In recent years, researchers have been developing new tools to evaluate attention and emotion regulation that do not entail developed speech. One such tool is eye tracking which provides a spatially and temporally accurate method for studying attention and emotional processing in preverbal infants (Leppänen, 2016). Eye tracking can be combined with an age-appropriate emotional attention disengagement paradigm (Peltola et al., 2018), to evaluate processing of emotional faces. It has been shown that infants have a strong bias toward faces (Leppänen, 2016) and that during the second half of first year they start to show heightened attentional preference for faces expressing fear (Peltola et al., 2008; Leppänen, 2016; Leppänen et al., 2018). The heightened preference for salient social cues, “fear bias,” is well-established in humans during infancy, and also among other social species (Leppänen and Nelson, 2012; Pritsch et al., 2017), rooting this predisposition to preferably attend to affectively salient stimuli over less salient in our evolutionary history. In infancy, fear bias is manifested as longer looking times to fearful vs. non-fearful faces and lower probability to disengage attention from fearful vs. non-fearful faces when attention is distracted by salient stimuli presented to the visual periphery (Leppänen, 2016; Bayet et al., 2017). Previous studies have connected the presence of fear bias in infancy to a more positive interaction between a mother and infant (de Haan et al., 2004) and more secure attachment style (Peltola et al., 2015), suggesting a supportive function of the normative bias for infant socioemotional development. Thus, the robustness and specificity of this age-typical fear bias reflects an important aspect of socioemotional information processing during the second half of first year, and individual differences may therefore represent altered salience or differential emotional processing.

In this study, we investigated whether infants prenatally exposed to sGC differ from non-exposed infants in terms of their attention disengagement probabilities from faces (i.e., neutral, happy, fearful, and scrambled non-faces) toward salient distractors (i.e., geometric shapes). More specifically, we were interested whether the sGC-exposed infants show different “fear bias” (i.e., the probability to disengage from fearful vs. non-fearful faces toward salient distractors) from non-sGC-exposed infants at the age of 8 months, as deviant fear bias may be an intermediate phenotype reflecting aberrant emotion processing. The assessment point for tracking fear bias was set to 8 months as developmentally the phenomenon is well tractable at this particular age, and also individual differences might be detectable at the time of its peak development during the second half of the first year.

## Materials and Methods

### Participants

The study sample comprised of *n* = 363 infants from the ongoing FinnBrain Birth Cohort Study (Karlsson et al., 2018) for whom a successful eye-tracking data (see a description of the pre-processing of the eye-tracking data below, and a detailed description of the sample in Kataja et al. (2020) at 8 months of age was available. No exclusion criteria related to birth phenotype were applied to the participants. None of the infants showed anomalies or developmental disorders by the age of 8 months. The eye-tracking measurements were conducted between 2013 and 2016 as part of the Child Development and Parental Functioning Lab study visit at the infant age of 8 months (8 months, ±2 weeks from the due date), along with infant temperament and mother-child interaction observations. Mothers gave informed consent on behalf of their infant. They were also informed about the study details and their option to withdraw from the testing at any time without providing a specific reason. The Ethics Committee of the Hospital District of Southwest Finland approved the study protocol. The study was conducted in full compliance with the Helsinki Declaration.

### Synthetic Glucocorticoid (sGC) Treatment

The mothers receiving sGC treatment for threatening prematurity were identified by automated electronic screening of all of the FinnBrain mothers’ hospital records for an indication of sGC treatment. Further indications for sGC administration and information on hospitalization and pregnancy were then collected for this sGC receiving subsample manually from the hospital electronic patient files. Further, the list of identified pregnancies was double-checked from The Finnish Medical Birth Register kept by the National Institute for Health and Welfare^1^. Altogether 129 (3.4%) out of the total 3,808 FinnBrain Cohort mothers received sGC treatment for threatening prematurity. All women were treated in the same tertiary hospital, which implies minor dispersion in diagnostics, reporting and clinical practices. Also, the Finnish Current Care guidelines for preterm delivery (Preterm delivery: Current Care guidelines, 2018) unify the practices. The current practice is to give two doses of 12 mg betamethasone administered intramuscularly 24 h apart.

Lastly, the ID list of sGC mother-child-dyads was compared with that of the subsample having attended the 8-month Child Development and Parental Functioning Lab study visit, resulting in 12 mother-child-dyads with sGC administration. The identification of these dyads was all done in retrospect, so that the selection of sGC families within the eye-tracking experiment was not biased. On average, the sGC children were born earlier and weighed less at birth than the rest of the study sample (see Table 1), factors that were considered in the subsequent analyses. All women received their sGC doses on two consecutive days. Regarding medication, 18 mothers had selective serotonin reuptake inhibitor (SSRI) medication at gestational week (GW) 14 and 20 mothers at GW 34. In the sGC treated group, one mother had SSRI medication and one had norepinephrine–dopamine reuptake inhibitor (NDRI) medication to treat depression during pregnancy. Of all women, *N* = 14 had another corticosteroid treatment either at GW 14 or at GW 34, and of the sGC treated group one had another corticosteroid (prednisolone) treatment during pregnancy (small dosage for ulcerative colitis). Of all mothers, *N* = 60 mothers had gestational diabetes. Sensitivity analyses were conducted by excluding the infants of these mothers (see Statistical analyses below).

**TABLE 1:** Characteristics of the study sample.

	**All (*N* = 363)**	**Treated (*N* = 12)**	**Non-treated (*N* = 351)**	***p***
Child sex (%, boys)	54.3%	58.3%	54.3%	0.798
Maternal age at birth [Mean(SD)]	30.75 (4.31)	29.67 (4.66)	30.78 (4.30)	0.382
	Mean (range)			
GW at birth	39.92 (7.86)	37.75 (6.28)	40.00 (6.58)	**<0.0001**
Birth weight	3592.56 (2580.00)	3304.17 (1865.00)	3602.45 (2580.00)	**0.030**
**Maternal questionnaires**				
SCL-90, GW 14	3.24 (4.09)	3.43 (2.49)	3.24 (4.14)	0.204
EPDS, GW 14	4.79 (4.16)	4.33 (3.14)	4.82 (4.19)	0.965
SCL-90, GW 24	4.16 (5.11)	4.39 (5.60)	4.16 (5.10)	0.789
EPDS, GW 24	4.76 (4.62)	4.17 (4.06)	4.78 (4.65)	0.770
SCL-90, GW 34	3.22 (4.63)	2.00 (1.79)	3.27 (4.69)	0.963
EPDS, GW 34	4.68 (4.65)	3.64 (1.91)	4.70 (4.71)	0.925
SCL-90, 3 months	2.47 (3.79)	0.78 (1.09)	2.53 (3.83)	0.185
EPDS, 3 months	3.84 (3.71)	1.56 (2.13)	3.90 (3.73)	**0.041**
SCL-90, 6 months	2.98 (4.24)	1.63 (1.60)	3.03 (4.28)	0.671
EPDS, 6 months	4.51 (4.48)	2.75 (1.83)	4.56 (4.53)	0.461

*GW, gestational weeks; SCL-90, Symptom Check List-90; EPDS, Edinburgh Postnatal Depression Scale. *p-*values refer to the comparisons between the treated vs. non-treated mothers. Bold values denote statistical significance at the *p* < 0.05 level.*

Regarding the timing of administration, the earliest phase in pregnancy was GW 27 + 5 days, the latest GW 35 + 1 day. The reasons behind suspected threat of preterm labor varied, with premature contractions accounting for the majority (nine premature contractions, one hemorrhage due to placenta praevia, one hepatogestosis with rising liver enzymes despite medication, and one fetus large for gestational age). In the sGC subsample, the earliest delivery took place at 34th GW + 3 days and the latest at 40th GW + 5 days. Seven (58%) out of the 12 children were born full term, i.e., at 37 GW or more.

### Eye-Tracking Assessments

During eye tracking, the infant sat on the parent’s lap at the distance of 50–70 cm from the eye tracker (EyeLink 1000+, SR Research Ltd., Toronto, ON, Canada). A sampling frequency of 500 Hz was used. Before every measurement, a five-point calibration procedure, with an audiovisual animation (i.e., a duck or a dog) sequentially presented in five locations on the screen, was used to assure the quality of the measurement. The calibration could be repeated before actual testing and also during measurement when necessary. Small breaks were allowed during measurement if needed. The eye-tracking laboratory was dimly lit and the researcher sat on an independent host computer next to the infant-parent dyad, but was separated by a curtain to avoid interference.

The overlap paradigm (Peltola et al., 2008) was used to study infant attention disengagement from a centrally presented face or a scrambled face control stimulus to a lateral distractor. Photographs of two different women portraying happy, fearful, and neutral faces together with scrambled face control pictures were shown. A set of 48 trials were presented, 12 trials per condition (each emotion and the control picture), comprising 18 photographs of each woman, and 12 scrambled face control pictures, in a semi-random order.

**FIGURE 1:**
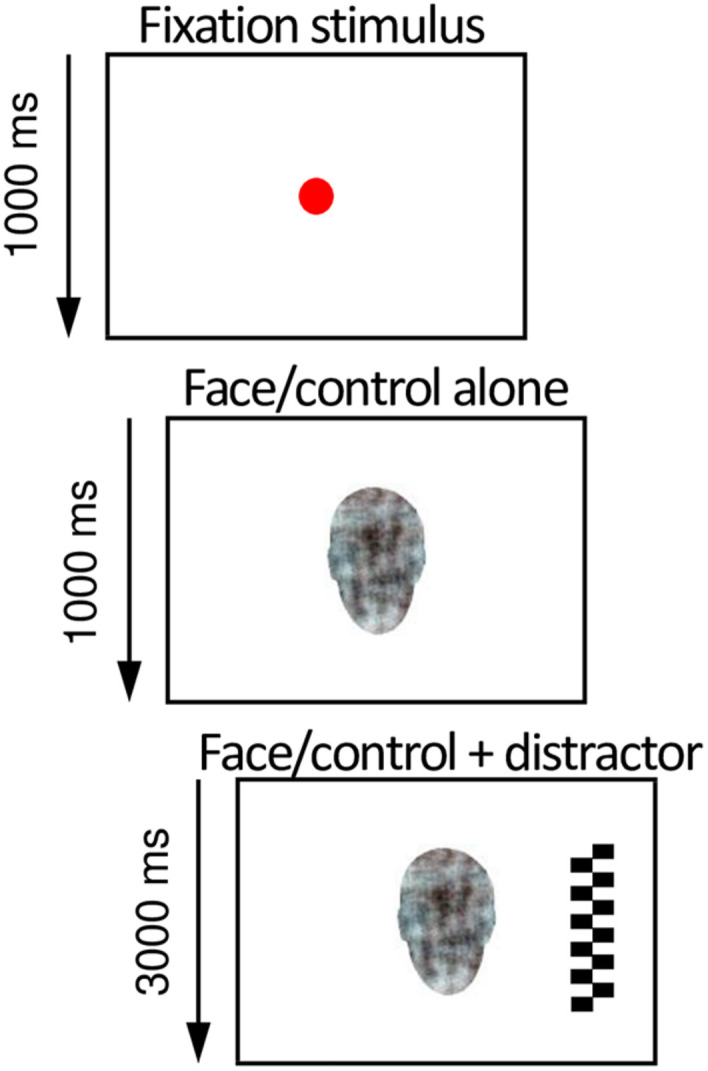
Illustration of the overlap paradigm used in the eye-tracking experiment to assess infant’s attention to social signals of emotion. After the infant looked at an animated fixation stimulus in the center of the screen (depicted here as a red circle), a face or a non-face pattern and subsequently a high-contrast lateral distractor were presented. The probability of attention disengagement from the central to the lateral stimulus was analyzed from the eye tracking data and used as a measure of attention to non-face patterns and neutral, happy, and fearful faces.

Before each trial, a fixation stimulus was shown to capture the attention of the infant to the center of the screen. Once the infant’s gaze was in the middle of the screen, the trial was presented by the researcher. First, a picture of a face (or a scrambled face control stimulus) was shown in the center of the screen for 1,000 ms. Then, a salient lateral distractor (checkerboard or circles) appeared on either the left or the right side of the face (a visual angle of 13.6°) for 3,000 ms, simultaneously with the face (Figure 1). One trial lasted for 4,000 ms. The sizes of the emotion-depicting pictures and distractor stimuli were 15.4°× 10.8° and 15.4°× 4.3°, respectively. The order of the central stimuli was semi-randomized, with a constraint that the same stimulus was not presented more than three times in a row. The lateral stimulus was selected and presented randomly for each trial.

### Preprocessing of Eye-Tracking Data

The trial data, comprising of timestamps for the onset times of central and lateral pictures and the xy coordinates of the participants’ gaze position (500 samples per second) were stored as text files, and analyzed offline using a library of Matlab scripts (Mathworks, Natick, MA, United States) (Leppänen et al., 2015). The following quality control criteria were used based on prior studies (Leppänen et al., 2015) to retain trials for the analysis. First, trials had to have sufficiently long fixation on the central stimulus (i.e., >70% of the time) during the time preceding gaze disengagement or the end of the analysis period (i.e., 1,000 ms from the appearance of the lateral distractor). Secondly, trials had to have a sufficient number of valid samples in the gaze data (i.e., no gaps >200 ms). Thirdly, trials had to have valid information about the eye movement from the central to the lateral stimulus (i.e., the eye movement did not occur during a period of missing gaze data).

First, the probabilities of disengagement (DPs) were calculated separately for each stimulus condition (i.e., neutral, happy, and fearful faces, and scrambled face control pictures). Then, to investigate the differences in infants’ fear bias, a fear bias score was calculated. Following (Yrttiaho et al., 2014) the “fear bias” was calculated by contrasting the fear condition (Fearful, FE) to the other face conditions (Happy, HA, Neutral, NE) using the following formula:

Fear bias=p(saccade/NE&HA)--p(saccade/FE).

### Covariates

#### Maternal Questionnaires

Maternal general anxiety and depressive symptoms during pregnancy were assessed at GW 14, 24, and 34 as well as at 3 and 6 months postpartum, using the anxiety subscale of the Symptom Checklist-90 (SCL-90) (Derogatis et al., 1973), and the Edinburgh Postnatal Depression Scale (EPDS) (Cox et al., 1987), respectively. Both showed good internal consistency, EPDS α = 0.82–0.89 and SCL-90 α = 0.85–0.90 in each assessment point.

#### Maternal Health, Birth, and Infant Characteristics

Information on maternal health status, infant due date, gestational age at birth, birth weight, and sex were collected from hospital records and the Finnish National Birth Register (see text footnote 1).

### Statistical Analyses

All statistical analyses were performed using IBM SPSS 25. First, differences in the eye-tracking measures [i.e., DPs for each stimulus condition (neutral, happy, fearful faces, and scrambled face control pictures) and Fear bias] were evaluated between the two groups (i.e., infants with vs. without antenatal sGC treatment) with Independent samples *t*-tests. Second, the sGC treatment main effect on infant fear bias was tested in a Multiple hierarchical linear regression model. Here, antenatal sGC treatment (yes/no) was entered on the first step, prematurity (birth < 37 gwks, yes/no), gestational age at birth, weight at birth, and infant sex on the second, and maternal depressive symptoms (EPDS sum) at 3 months postpartum on the third step.

Moreover, sensitivity analyses were conducted by excluding infants of mothers with either antidepressant (SSRI/SNRI/NDRI) medication or other corticosteroid treatment during pregnancy or maternal gestational diabetes from the analyses. These exclusions did not alter the main results, and the results are therefore reported for the whole sample.

## Results

The characteristics of the study sample are displayed in Table 1. Out of 363, 8-month-old infants, 12 had been treated with sGC *in utero* and 351 had not. All except one mother were administered with two doses of 12 mg betamethasone 24 h apart. The exception had the two doses within the period of 24 h.

No between-groups difference was found regarding maternal age (Table 1). As expected, average gestational age at birth was smaller in the sGC group and sGC newborns also presented with lower weight at birth (Crowther et al., 2019). Gestational age at birth, prematurity, and birth weight were therefore controlled in further analyses.

No major differences were found in maternal anxiety or depressive symptoms during pregnancy or early postpartum (assessed with SCL-90 and EPDS, respectively), with the exception of EPDS scores being higher among the non-treated mothers 3 months postpartum (*p* = 0.041; Table 1). Maternal depressive symptoms at 3 months postpartum were controlled for in further analyses.

In the overlap paradigm, across the whole sample, the disengagement probabilities (DPs) were highest for the control, scrambled non-face pictures (*M* = 0.80, SD = 0.21), intermediate for the neutral and happy faces (*M* = 0.61, SD = 0.26 and *M* = 0.61, SD = 0.26, respectively), and lowest for the fearful faces (*M* = 0.46, SD = 0.28). All paired comparisons reached *p*-values <0.001, except for neutral and happy faces *p* = 0.57. An age-typical fear bias (*M* = 0.15, SD = 0.20) was observed across the whole sample with infants disengaging their attention less frequently from fearful vs. happy and neutral faces (*p* < 0.001) as expected.

**FIGURE 2:**
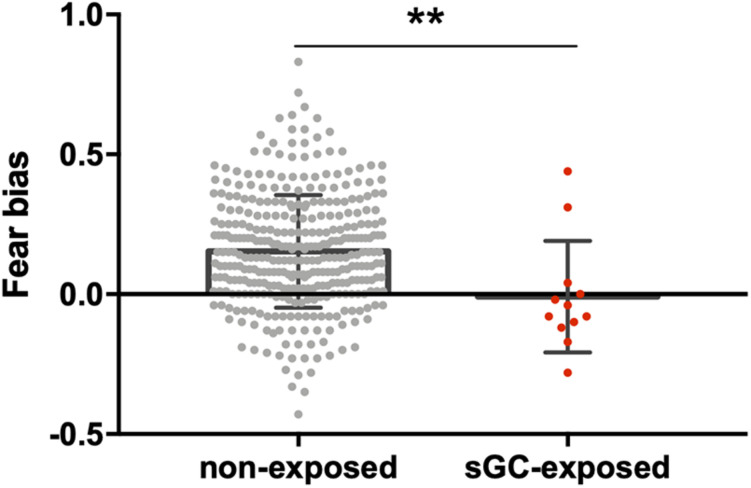
Comparison between 8-month-old infants with prenatal sGC treatment exposure (sGC-exposed) and without treatment exposure (non-exposed). The sGC group showed a statistically significant reduction in fear bias. **p < 0.01.

When comparing the infants with and without antenatal sGC treatment exposure, the two groups did not significantly differ in their DPs in the control, neutral, happy, or fearful conditions (all *p*-values >0.16, Table 2). However, a significant difference was observed in the fear bias between the groups (see Table 2 for group means and Figure 2). The infants with antenatal sGC treatment exposure did not show an age-typical fear bias (*M* = −0.01, SD = 0.20), whereas the non-treated infants did (*M* = 0.15, SD = 0.20), *t*(360) = 0.358, *p* = 0.007, 95% CI[0.04, 0.28].

**TABLE 2:** Eye-tracking results (mean, SD).

	**All *N* = 363**	**sGC treated *N* = 12**	**Non-treated *N* = 351**	***p***	***95% CIs of the difference***		***Cohen’s d***
DP Control	0.80 (0.21)	0.88 (0.16)	0.80 (0.21)	0.161	−0.21	0.03	0.46
DP Neutral	0.61 (0.26)	0.57 (0.31)	0.62 (26)	0.554	−0.11	0.20	0.16
DP Happy	0.61 (26)	0.51 (0.33)	0.61 (0.25)	0.185	−0.05	0.25	0.34
DP Fearful	0.46 (0.28)	0.55 (0.34)	0.46 (0.28)	0.288	−0.25	0.07	0.28
Fear bias	0.15 (0.20)	−0.01 (0.20)	0.15 (0.20)	**0.007**	0.04	0.28	0.80

*DP, disengagement probability. Bold values denote statistical significance at the *p* < 0.05 level.*

In the linear regression model, antenatal sGC treatment was a significant predictor of fear bias (standardized β = −0.145, *p* = 0.017) after controlling for prematurity (*p* = 0.33), gestational week (*p* = 0.35) and weight (*p* = 0.85) at birth, child sex (*p* = 0.77) and maternal depressive symptoms at 3 months postpartum (*p* = 0.05). The model explained 2.2% of the variance in fear bias [*R*^2^ = 0.04, *F*_(__6_,_31__2__)_ = 2.20, *p* = 0.043]. See Table 3 for the full model.

**TABLE 3:** Hierarchical multiple regression for Fear bias for the whole sample.

	***R*^2^**	***ΔR^2^***	***Unstandardized β***	***Standardized β***	***Sig.***	***F for change in R^2^***
Step 1	0.023**	0.020**				7.404**
Antenatal sGC			−0.178**	−0.151**	0.007	
Step 2	0.029	0.014				0.500
Antenatal sGC			−0.180*	−0.152*	0.012	
Prematurity			−0.071	−0.067	0.337	
GW at birth			0.012	0.080	0.273	
Weight at birth			5.50	0.013	0.839	
Infant sex			−0.009	−0.022	0.693	
Step 3	0.038^†^	0.022^†^				3.741^†^
Antenatal sGC			−0.171*	−0.145*	0.017	
Prematurity			−0.073	−0.068	0.325	
GW at birth			0.010	0.068	0.352	
Weight at birth			2.00	0.005	0.853	
Infant sex			−0.006	−0.016	0.771	
EPDS sum 3 months			0.006^†^	0.109^†^	0.054	

sGC, synthetic glucocorticoids; GW, gestational weeks; EPDS, Edinburgh Postnatal Depression Scale.

**p < 0.01.

*p < 0.05.

*^†^*p*< 0.10.*

## Discussion

In this study, we investigated the associations between exposure to prenatal synthetic glucocorticoid (sGC) treatment and emotion processing at 8 months using eye tracking. We aimed to investigate the effects of exposure on a well-established infant phenotype, namely fear bias, soon after its emergence during the second half of the first year. Deviances in the processing of affectively salient cues, and specifically negative information, may represent an intermediate phenotype of later emotion regulation difficulties (Fox and Beevers, 2016). We found that sGC exposed infants, in comparison with non-exposed infants, did not show an age-typical bias for fearful vs. non-fearful facial expressions (e.g., Peltola et al., 2009; Nakagawa and Sukigara, 2012). The sGC treated infants did not differ from the non-treated in their general disengagement probability when viewing different faces and distractors, but the impact of specifically the fearful faces vs. other faces on attention disengagement was low, i.e., leading to low fear bias, which is atypical at this age.

Prioritized processing of fear or threat is a biologically conserved component of attention due to its clear implication for survival and well-being (Leppänen and Nelson, 2012; Pritsch et al., 2017). A heightened preference for fear emerges at the time of increased autonomy during infancy, both among humans and other social species, and is accompanied with other fear-related behaviors such as fear of strangers and heights (Leppänen and Nelson, 2012; Pritsch et al., 2017). Previous studies have found fear bias in infancy to be supportive for infant socioemotional development, possibly through more positive dyadic interaction patterns and attachment between the infant and caregiver (de Haan et al., 2004; Peltola et al., 2015). Our study suggests that sGC exposure may profoundly alter this infant-typical attention pattern. Thus, it is possible that sGC treatment influences infant neurodevelopment and specifically the emotional processing by altering the maturation of the related limbic brain networks.

In humans, associations between sGC treatment and later neurodevelopmental, emotional and behavioral problems in offspring have been reported by several studies (Cartier et al., 2016; Wolford et al., 2019; Räikkönen et al., 2020). Altered fear processing may be one of the early signs of deviant emotional processing after sGC treatment, as deviances in attention biases for negative information have been consistently connected with risk of psychopathology, especially if combined with genetic or environmental risk (Bar-Haim et al., 2007; Fox and Beevers, 2016). Currently, however, research on the developmental trajectory of fear bias beyond infancy is limited. Two previous studies, using a comparable attention-distraction paradigm, reported a decline in fear bias toward the third year of life suggesting that the salience of fearful faces on attention processes is especially high during the second half of first year (Nakagawa and Sukigara, 2012; Peltola et al., 2018). Thus, in our study the low fear bias among the sGC treated infants may reflect an altered pace of development of the fear processing systems.

The evolutionary nature of fear bias posits that it is plastic to environmental factors, enabling changes according to environmental needs. According to the stress acceleration hypothesis early-life factors such as adverse caregiving experiences might accelerate the maturation of emotion circuits as an adaptation of the system (Callaghan and Tottenham, 2016). Indeed, previous studies, including our own, using the emotional overlap paradigm have shown that the infant fear processing systems are sensitive to early-life exposures, including maternal psychological distress (Forssman et al., 2014; Nolvi et al., 2016; Morales et al., 2017; Kataja et al., 2019) and caregiving (Callaghan and Tottenham, 2016). For example, both maternal depressive and anxiety symptoms have been found to associate with higher fear bias in infants (Forssman et al., 2014; Morales et al., 2017; Kataja et al., 2019, 2020). Given that these findings point in the opposite direction compared with the results of the current study, i.e., showing an association between higher maternal symptoms of distress and higher fear bias in their infants, we may speculate that the fear processing systems of the sGC treated infants do not reflect an accelerated but delayed pace of development. Different types of exposures may have a differential role in shaping infant fear (face) processing which is yet to be determined.

Importantly, the prevalence of any mental, emotional, and behavioral disorder has been found to be higher in sGC-exposed as compared to non-exposed children (Wolford et al., 2019). Parents have also reported that sGC-exposed children present delays in their development and personal-social skills, independently of prematurity (Wolford et al., 2019). As we observed a reduction in age-typical fear bias in sGC infants this may also be an indicator for a delayed development of the neuronal circuits underlying emotional processing. This, then, would lead to a low fear bias at the time of its typical peak during development. Other studies have shown that the lack of age-typical fear bias at 7 months is associated with insecure attachment between the infant and the caregiver later in life (Peltola et al., 2015), connecting difficulties in the formation of a secure attachment and reaching age-typical developmental milestone in fear processing. Longitudinal follow-up of the same children is needed to better understand the nature and significance of the results.

If the observed reduction in fear bias in sGC infants supposedly reflects altered maturity of the brain circuits underlying emotional regulation, it is relevant to point out that sGC can induce structural and functional changes in brain regions such as the amygdala or nucleus accumbens. Changes in brain areas important for emotion generation and regulation have been observed in a rodent model that mimics prenatal sGC administration to pregnant women. For example, a rodent model that mimics prenatal sGC administration to pregnant women, presents molecular and anatomical changes in several brain regions sensitive to glucocorticoids and important for emotion attention interactions such as the prefrontal cortex, hippocampus, amygdala, and nucleus accumbens (Leão et al., 2007; Oliveira et al., 2012; Rodrigues et al., 2012; Cartier et al., 2016). Moreover, sGC-exposed animals reportedly have prominent emotional deficits later in adulthood, such as heightened fear responses (Borges et al., 2013b) and depressive-like behaviors (Oliveira et al., 2006; Borges et al., 2013a; Soares-Cunha et al., 2014) suggesting a role for prenatal glucocorticoids in programming neurodevelopmental disorders. The observed increase in fear response in rodents prenatally exposed to sGC is particularly interesting in the context of our findings, suggesting that sGC can alter the specific neuronal networks involved in fear processing.

However, the literature on prenatal sGC effects in the human brain is scarce. One study has shown that exposure to sGC dexamethasone in the first trimester of gestation is associated with structural changes in the amygdala in humans later in life (van’t Westeinde et al., 2020). Other study has shown that prenatal sGC treated children had a thinner rostral anterior cingulate cortex (rACC), however, in the absence of emotional/behavioral alterations. Since rACC thinness is associated with affective problems, authors postulated that sGC-associated changes increase vulnerability to mental problems later in life (Davis et al., 2013). Though associations between sGC treatment and later emotional and behavioral problems in human offspring have been reported by different studies (Khalife et al., 2013; Wolford et al., 2019; Räikkönen et al., 2020), others have found no association between prenatal sGCs and later adverse outcomes (Dalziel et al., 2005; Hirvikoski et al., 2008; Davis et al., 2013; Stutchfield et al., 2013; Alexander et al., 2016). Therefore, additional research is needed to reveal the circumstances under which prenatal sGC treatment may be a significant predictor for offspring development. For instance, the timing and dose of treatment as well as various postnatal factors are all likely crucial in determining later effects on the offspring (Cartier et al., 2016).

Importantly, this study also raises a clinically relevant question of the optimization of the current guidelines for sGC administration due to preterm labor. Only 2–10% of singleton pregnancies assessed due to preterm contractions end up in delivery within the next 7–14 days (Fuchs et al., 2004; Wing et al., 2017). Ultrasound and different biomarkers have been developed to increase reliability of predicting preterm labor, but no consensus has been obtained regarding their use. This is particularly important to refer as in our study, 50% of sGC-exposed individuals were born full term. A Dutch prospective cohort study also showed that 20–60% of sCG treatments are prescribed to low risk women, of whom only 1–3% give birth within the next 7 days (Wilms et al., 2015). In an Australian study with 17 754 subjects, the rate of antenatal sGC administrations was shown to be on the rise, although the rate of preterm deliveries remained stable over time (Polyakov et al., 2007). Even when the risk is deemed as substantial and sGC is thus administered, approximately one third to a half of pregnancies proceed to term (Polyakov et al., 2007). This highlights the need to pursuing research to identify factors that are accurate and true predictors of preterm labor, in order to be able to select the pregnancies that would truly benefit from sGC administration from the perspective of child’s future health and development.

### Conclusion and Limitations

In sum, our study shows that prenatal sGC exposure induces alterations in fear processing in 8-month-old infants, which suggests altered emotional development. Further studies are needed to unravel if these changes are associated with alterations in emotional and/or social development later in life. While the functional significance of our findings and their predictive value for later emotional/developmental problems remain subject to further investigation, we may state that prenatal sGC treatment appears to be connected to a well-established feature of infant attention, i.e., fear bias. This bias is typically high during the second half of the first year, and is accompanied with the normative emergence of other fear-related behaviors (Leppänen and Nelson, 2012). As a limitation, despite that we controlled for several possible confounding factors there may still be other, either mother- of child-related factors, that have led to sGC treatment as well as deviances in infant fear processing. For instance, we could not test the possible sex differences in the effects of sGC treatment due to our small sample size in the index group despite that the effects may also be sex-specific. Replication as well as longitudinal studies with larger sample sizes are urgently needed in order to understand the implications of prenatal sGC-induced alterations in infant social-emotional processing and development. Further, maternal state continues to influence infant development after delivery. A limitation to our study is that we did not assess maternal symptoms at the time of eye-tracking at 8 months of infant age. However, we measured the symptoms at 6 months postpartum and used it as a proxy of the maternal psychological condition at the time of infant fear bias assessment.

## Data Availability Statement

The datasets presented in this article are not readily available because due to Finnish Federal Legislation, the research data cannot be made available online, but data can potentially be shared with Material Transfer Agreement. Requests can be directed to the Board of the FinnBrain Birth Cohort Study. Requests to access the datasets should be directed to LK, linnea.karlsson@utu.fi.

## Ethics Statement

The studies involving human participants were reviewed and approved by the Ethics Committee of the Hospital District of Southwest Finland. Written informed consent to participate in this study was provided by the participants’ legal guardian/next of kin.

## Author Contributions

E-LK participated in the planning of the study design, took care of the eye-tracking data collection, analyzed the data, and drafted the first manuscript version. AR drafted the first manuscript version and were involved in data analysis. LK and EE participated in the planning of the study design and revised the manuscript. TH contributed to the building of the eye-tracking paradigm and data managing. NMS participated in the planning of the study design and sample acquisition, conducted sample screening from hospital records, and revising of the manuscript. RK, SN, and NS participated in the revising of the manuscript. HK planned the study design, provided the funding for the study, and revised the manuscript. All authors approved the final version of the manuscript for submission.

## Conflict of Interest

The authors declare that the research was conducted in the absence of any commercial or financial relationships that could be construed as a potential conflict of interest.

## Publisher’s Note

All claims expressed in this article are solely those of the authors and do not necessarily represent those of their affiliated organizations, or those of the publisher, the editors and the reviewers. Any product that may be evaluated in this article, or claim that may be made by its manufacturer, is not guaranteed or endorsed by the publisher.

## Footnotes

1
www.thl.fi
